# Serological prevalence of viral agents that induce reproductive failure in South Korean wild boar

**DOI:** 10.1186/s12917-015-0396-7

**Published:** 2015-03-26

**Authors:** Hye-Young Jeoung, Seong-In Lim, Jae-Jo Kim, Yoon-Young Cho, Yong Kwan Kim, Jae-Young Song, Bang-Hun Hyun, Dong-Jun An

**Affiliations:** Viral Disease Division, Animal and Plant Quarantine Agency, Anyang, Gyeonggi-do 430-824 Republic of Korea

**Keywords:** Aujeszky’s disease virus (ADV), Encephalomyocarditis virus (EMCV), Porcine parvovirus (PPV), Serological survey, Wild boar

## Abstract

**Background:**

Viral agents associated with reproductive failure such as Aujeszky’s disease virus (ADV), encephalomyocarditis virus (EMCV), and porcine parvovirus (PPV) have also been identified in European wild boar. To screen for the presence of antibodies against ADV, EMCV, and PPV from wild boar (*Sus scrofa*) in South Korea, 481 serum samples were collected from wild boar hunted between December 2010 and May 2011.

**Results:**

Of the 481 serum samples tested, 47 (9.8%) and 37 (7.7%) were seropositive for ADV and EMCV antibodies, respectively, based on a neutralization test (VNT), and 142 (29.5%) were seropositive for PPV antibodies based on a hemagglutination inhibition (HI) test.

**Conclusions:**

This was the first survey to identify the seroprevalence of the three major viruses associated with reproductive failure in the wild boar population of South Korea. Wild boar may act as a reservoir for many viruses that cause infectious diseases in domestic pigs. Thus, strict prevention and control measures, such as continuous wildlife disease surveillance and strategic methods of downsizing the population density, should be implemented to prevent disease transmission from wild boar to domestic pigs.

## Background

Wild boar (*Sus scrofa*) may act as a reservoir for many infectious pathogens, such as the causative agents of zoonoses and livestock infectious diseases, and the domestic pig and wild boar share numerous common pathogens [1]. In Europe, it was reported that wild boar may constitute a reservoir for many disease that affect domestic pigs [1,2]. Viral agents associated with reproductive failure such as Aujeszky’s disease virus (ADV), encephalomyocarditis virus (EMCV), porcine parvovirus (PPV), porcine circovirus type 2 (PCV2), and porcine reproductive and respiratory syndrome virus (PRRSV) have also been identified in European wild boar [2-5]. In South Korea, the wild boar population has increased continuously during the last 30 years because of a lack of predators and competitors [6]. This growing population could also increase the risk of Korean wild boar acting as a reservoir for various infectious agents and spreading diseases. Recent studies have detected pathogens in South Korean wild boar, including classical swine fever virus (CSFV), PRRSV, hepatitis E virus, and trichinella [6-9]. However, few studies have investigated the possible links between disease outbreaks and intra-species disease transmission from wild boar to domestic pigs in South Korea. Aujeszky’s disease, which causes mummification and abortion in pregnant sows, is an economically important disease for the pig industry worldwide and many countries have implemented national programs to eliminate this disease [10-13]. EMCV infection of swine can cause severe economic losses on pig farms and is known to be a cause of mortality in young pigs, acute myocarditis, and reproductive failure in sows [14]. PPV is common in domestic swine, where it is implicated in early fetal death, stillbirth, and weak birth [4]. The present study tested for antibodies against important viral agents that induce reproductive failure, such as PPV, ADV, and EMCV, in South Korean wild boar and examined the possibility of disease transmission between domestic pigs and wild boar. The results may facilitate the development of effective wild boar disease control programs.

## Results and discussion

The overall seropositivity rates were 9.8% (47/481) for ADV, 7.7% (37/481) for EMCV, and 29.5% (142/481) for PPV (Table 1). However, there were no correlations between the prevalence rates of these diseases and the geographical origins of the samples because only a small number of samples were collected and their geographical distribution was very limited, which was possibly biased by hunting area restrictions. No animals were infected simultaneously with all three viral pathogens. However, antibodies against ADV (32-fold) and EMCV (64-fold) were both detected in one sample from Gyeonggi.

**Table 1:** **Seropositive antibody titers against Aujeszky’s disease virus (ADV), encephalomyocarditis virus (EMCV), and porcine parvovirus (PPV) in wild boar from five South Korean provinces**

**Province**	Antibody titers against ADV/EMCV/PPV*							
	**8**	**16**	**32**	**64**	**128**	**256**	**512**	**Total**
Gangwon	-^a^/-^b^/1^c^	5/-/1	1/0/11	1/3/13	3/0/2	0/2/5	0/0/1	10/5/34
Gyeonggi	−/−/2	12/-/2	5/1/8	2/6/6	0/5/3	0/4/1	0/0/2	19/16/24
Chungchung	−/−/3	3/-/9	1/2/3	1/1/11	1/2/4	0/3/6	0/0/3	6/8/39
Gyeongsang	−/−/1	1/-/4	0/3/7	4/0/3	1/0/6	0/0/1	0/0/1	6/3/23
Jeonra	−/−/0	6/-/2	0/0/2	0/1/9	0/1/2	0/3/2	0/0/5	6/5/22
Total	−/−/7	27/-/18	7/6/31	8/11/42	5/8/17	0/12/15	0/0/12	47/37/142

Numbers of samples that tested positive for antibodies against ^a^ADV/^b^EMCV/^c^PPV.

Samples were considered positive if the titers were ≥1:16 for ADV, ≥1:32 for EMCV, and ≥1:8 for PPV.

*A neutralization test was used to detect ADV and EMCV antibodies, and a hemagglutination inhibition was performed to test for PPV antibodies.

- : Negative for antibodies against ADV or EMCV.

Disease control among wild boar is a primary concern of those involved with wildlife and domestic livestock because the wild boar population has become susceptible to pathogens that may serve as a reservoir for domestic pig disease viruses. Pathogens that are responsible for reproductive failure in domestic pigs could have similar effects in wild boar [2]. The transmission of viruses that cause reproductive failure from wild boar to domestic pigs may have severe effects on piglet production, with huge economic losses on pig farms. In South Korea, direct contact between wild boar and domestic pigs may rarely occur because domestic pigs are usually reared within enclosed farm facilities. However, some farmers raise their pigs near mountains with rough fences and pigs may escape from their pens. Wild boar have started to invade residential areas to search for food and there are increasing reports of damage by wild boar in residential areas, including the outskirts of cities [6]. Compared with other countries with less dense human and animal populations, such as the USA and Canada, contacts between wild boar and domestic pigs could occur more easily in South Korea. It is possible that domestic pigs and wild boar could come into contact and share pathogens, which is supported by the detection of CSFV and PRRSV in wild boar samples [6,15]. Two CSFV strains (YC11WB and PC11WB) classified as subgroup 2.1b were isolated from Korean wild boar in 2011 [15]. Of 267 wild boar samples analyzed, four (1.5%) were also positive for PRRSV antibodies and eight (3.0%) were positive for European type 1 and North American type 2 PRRSV antigens. The nucleotide sequences of the type 1 PRRSV ORF7 had 96.1–98.4% shared identity with PRRSV from domestic pigs in Korea, and the sequences of type 2 PRRSV ORF7 had 100% shared identity with PRRSV strain VR-2332, which is the prototype North America strain [6]. ADV, EMCV, and PPV antibodies have also been reported from wild boar populations in Europe and the USA [16-18].

The prevalence of EMCV antibodies in domestic pigs from South Korea was found to be 9.1% (301/3315) [19]. In the present study, the prevalence of EMCV antibodies (7.7%) in Korean wild boar was similar to that in domestic pigs. The present study also detected PPV antibodies in 142 (29.5%) Korean wild boar serum samples, which suggests that PPV has a widespread distribution in the wild boar population.

The relatively high prevalence of antibodies against ADV in Korean wild boar is surprising because the domestic pig population has been free of ADV since 2010 according to the Korea Animal Health Integrated System (KAHIS) (http://www.kahis.go.kr), which stores animal disease surveillance results submitted by the local and national governments of South Korea. KAHIS analyzed 137,101 swine serum samples in 2010, 148,576 samples in 2011, and 146,006 samples in 2012, and all samples were negative for ADV antibodies. In the USA, it is estimated that the large population of wild boar is a potential source for re-infection where the prevalence of ADV may exceed 60% [20]. In the Czech Republic, it was reported that ADV infection of domestic pigs occurred after contact with wild boar [5]. Therefore, the domestic and captive wild boar population should be separated from the wild and feral pig population by appropriate biosecurity measures to prevent transmission of ADV in South Korea.

## Conclusion

In conclusion, we found no evidence of the transmission of infectious agents between domestic pigs and the wild boar population, but viruses associated with reproductive failure had a relatively high prevalence in the wild boar population. Contacts between pigs and wild boar are infrequent events, but several examples have been reported, which support the link between open air or backyard pig production and the risk of disease transmission at the pig-wild boar interface, such as classical swine fever in Germany [21] and African swine fever in Sardinia [22]. Effective disease control in wild boars demands continuous serological surveys to establish surveillance programs. Backyard pig vaccination campaigns to combat ADV, PPV, and EMCV may also have major roles in preventing transmission at the pig–wild boar interface.

## Methods

### Wild boars serum samples

Blood samples were collected from wild boar hunted and killed as part of a classical swine fever (CSF) eradication campaign undertaken in South Korea between December 2010 and May 2011. Most of the samples were obtained from adult boar, but information was not available on their age and sex. After shooting, the wild boar blood samples were collected in sterile tubes and transported immediately to the laboratory. The serum samples were harvested by centrifugation at 1,500 × *g* for 15 min at 4°C and stored at −20°C until analysis.

### HI Assay and VN test

The 481 serum samples originated from five provinces: Gangwon (n = 79), Gyeonggi (n = 153), Chungchung (n = 102), Gyeongsang (n = 86), and Jeonra (n = 61). The sera were analyzed using a neutralization test (VNT) for two viruses (ADV and EMCV) and by a hemagglutination inhibition (HI) test for PPV, as described previously [19,23,24]. Samples were considered positive if the titers were ≥1:16 for ADV, ≥1: 32 for EMCV, and ≥1:8 for PPV [19,25,26].
